# Are we ready for a sustainable approach? A qualitative study of the readiness of the public health system to provide STI services to the key populations at risk of HIV in Bangladesh

**DOI:** 10.1186/s12913-023-09996-2

**Published:** 2023-09-11

**Authors:** Gorkey Gourab, Golam Sarwar, Mohammad Niaz Morshed Khan, A M Rumayan Hasan, Samira Dishti Irfan, Tarit Kumar Saha, Lima Rahman, A. K. M. Masud Rana, Sharful Islam Khan

**Affiliations:** 1 Programme for HIV and AIDS, Infectious Diseases Division, International Centre for Diarrhoeal Diseases Research, 68 Shaheed Tajuddin Ahmed Sarani, Mohakhali, Dhaka 1212 Bangladesh; 2 Graduate School of Biomedical Sciences, Hiroshima University, Hiroshima, Japan; 3 Universal Health Coverage, Health System and Population Studies Division, International Centre for Diarrhoeal Diseases Research, 68 Shaheed Tajuddin Ahmed Sarani, Mohakhali, Dhaka, Bangladesh; 4 Institute of Public Health (IPH), Dhaka, Bangladesh; 5 Ministry of Health and Family Welfare, 68 Shaheed Tajuddin Ahmed Sarani, Mohakhali, Dhaka, Bangladesh; 6 HIV/AIDS Programme, Health, Nutrition and HIV/AIDS Sector, Save the Children, House 35, Road 43, Gulshan-2, Dhaka, Bangladesh

**Keywords:** HIV, Sustainability, Readiness, STI, Key populations, Service availability and readiness

## Abstract

**Introduction:**

In Bangladesh, sexually transmitted infection (STI) services are available for all populations in public health facilities. However, STI services for key populations (KPs) at risk of HIV need specifically designed approaches that are predominantly administered to KPs through donor-supported service centers operated by non-government organizations (NGOs) and community-based organisations (CBOs). However, the steady decline in donor funding warrants a sustainable transition of STI services for the KPs into public health facilities. This article aimed to explore the service availability and readiness of public health facilities to provide STI services for the KPs.

**Methods:**

This qualitative study explored the service availability and readiness of public health facilities in three districts of Bangladesh by adapting the Service Availability and Readiness Assessment tool. We conducted 34 in-depth interviews,11 focus group discussions with KPs, and 29 key-informant interviews with healthcare providers, researchers, programme implementers and policy planners, in addition to series of direct observations at the public healthcare facilities. Data were analysed through thematic analysis, and categorised in relation to the WHO building blocks.

**Results:**

This study revealed that the public health system was generally not ready to serve the KPs’ needs in terms of providing them with quality STI services. The ‘service delivery’ component, which is the most crucial facet of the public health system, was not ready to provide STI services to KPs. Findings also indicated that health workforce availability was limited in the primary and secondary healthcare layers but adequate in the tertiary layer, but needed to be oriented on providing culturally sensitised treatment. Counseling, an essential component of STI services, was neither ready nor available. However, health information systems and a few other components were partially ready, although this warrants systematic approaches to address these challenges.

**Conclusion:**

The findings show that public health facilities are yet to be fully ready to render STI services to KPs, especially in terms of service delivery and human and health resources. Therefore, it is not only integral to mobilize communities towards the uptake of public health services, but health systems need to be prepared to cater to their needs.

**Supplementary Information:**

The online version contains supplementary material available at 10.1186/s12913-023-09996-2.

## Introduction

A health system is a tangible yet abstract entity consisting of “all organisations, people, and actions whose purpose is to promote, restore, and maintain health” [1]. The public health system refers to all components contributing to the delivery of health services within government authority [2, 3]. However, the public health system of many low- and middle-income countries (LMIC), including Bangladesh, adopts pluralistic as opposed to monopolistic approaches [3–5].

The health system of Bangladesh encompasses four components including public, private, non-government organisations (NGOs) and donors. In addition, like other LMICs, informal health service providers represent a substantial niche in the national health system [6]. The government is accountable for the stewardship of their respective health system. Therefore, the government could create a pathway for an equitable health system, so that health service providers are capacitated to deal with vulnerable, marginalised populations with greater need of accessible health services, such as key populations (KPs) at risk of HIV. It is worth noting that KPs are disproportionately affected by the HIV and STI burden, as illustrated by the local evidence. As of 2022, over a third (36%) of the newly diagnosed HIV cases belong to the KPs, thus making them the most prominent group of PLHIV compared to Rohingya refugees, migrants and other general populations [7]. Similarly, 5.7% of all of the KPs combined tested positive for active syphilis according to the latest surveillance findings for KPs in Bangladesh [8].

The existing targeted HIV prevention interventions for KPs in Bangladesh follow a community-based and peer-led approach, which operates as a parallel structure to public health systems. All activities are operated through static Drop-in Centers (DICs) and primarily depend on external resources, and are implemented by the NGOs. KPs include males who have sex with males (MSM), people who inject drugs (PWID), transgender women (known as *hijra*), and female sex workers (FSW). To elaborate, the HIV interventions have always been dependent on funding from international donors since their inception in 1995. Given the socially stigmatized and criminalized nature of KP groups, they preferred seeking healthcare from these DICs as opposed to mainstream healthcare facilities. However, throughout the years, the funding landscape has deteriorated for countries like Bangladesh which are gradually migrating towards becoming a middle-income country, thus threatening the sustainability of donor-funded healthcare. Therefore, since sustainable service delivery through DICs is a major challenge, sustainable strategies should ideally be embedded into project implementation to support the HIV response even after donor funding is exhausted [9].

In this context, it is integral to prepare the mainstream healthcare setup, particularly the public healthcare facilities, to become the first line of care for HIV and STI services for these populations. However, before preparing these facilities, it is crucial to understand the existing challenges of the public health system in terms of catering to the needs of these populations. In a similar vein, donors constantly indicate that integrating synergistic services, health systems strengthening and utilisation of health systems are necessary for the HIV response [10–13]. Moreover, a recent information note by the Global Fund emphasised that “countries remain the major investors in their own health systems” and encouraged countries to leverage their respective public health systems [14]. Moreover, various studies have indicated that rather than relying on the other components of a pluralistic health system, it would be more ideal to effectively utilise the public health system as a key player to ensure that patients’ needs are met [15–17].

However, it is generally claimed that the public health system is not ready to serve the needs of the KPs in culture-sensitive ways. To devise a sustainable and resilient approach to provide STI services, which include diagnosis and treatment of gonorrhea, syphilis, chlamydia, trichomoniasis, pelvic inflammatory disease (PID), genital herpes, bacterial vaginosis, and scabies to the KPs within the public health system, it is important to comprehensively analyse whether the public system is to serve the KPs in its existing state. Till date, no systematic research had been conducted to understand the readiness of the public health system in Bangladesh to deliver STI services to the KPs that are appropriately tailored to diverse needs of the KPs in Bangladesh. Hence, this study aims to understand and explore the readiness of public health systems to provide STI services to KPs utilising an adapted version of ‘The WHO Health Systems Framework’ [1].

### The WHO Health Systems Framework

The systems theory, which underscores the “importance of understanding the complete system and the underlying interactions of all the forces that make up that system”, is often utilised to understand and analyse a health system [18]. The application of this theory (i.e. systems thinking), known as systems analysis, helps visualise systems from broader perspectives including seeing “overall structures, patterns and cycles in systems”, rather than merely individual components of the system [19, 20]. Several recent studies have utilised systems thinking to understand health issues such as the control of tobacco intake, obesity and tuberculosis [21–23]. WHO indicates that systems thinking can help in understanding, pinpointing and addressing health system challenges, and therefore it is a crucial component to understanding any health system [18]. Considering the importance of the application of systems thinking, in 2007, WHO introduced ‘The WHO Health Systems Framework’, where the health system is analysed using six different groups or blocks [1].

WHO indicates that systems thinking can not only help decipher the complexity of an entire health system, but mainstreaming a stronger health system thinking perspective will help accelerate the health system strengthening process. Despite increased health system investments, very few countries have updated information about the availability of health systems, which could be useful for assessing the “readiness” of health systems to administer the availability and quality of health services. In light of this issue, several health systems models were developed to explore health service quality. Health systems frameworks of this nature could be used to address institutional and structural health systems challenges. For instance, WHO recommends the use of ‘Service Availability and Readiness Assessment (SARA)’, an assessment tool that is specifically designed to monitor and evaluate the service availability and readiness of health facilities [24]. It is a systematic survey consisting of tracer indicators of service availability and readiness [24], thus generating insights for improved health status and better outcomes. This tool is also recommended in facilitating HIV intervention scale-up efforts by assessing their readiness to deliver key interventions [24].

However, there is a lack of qualitative tools or framework to understand the readiness of a health system. Similarly, a qualitative framework to understand public health system readiness, especially for HIV and STI-related services for KPs is also absent. Hence, to understand the readiness of the public health systems through qualitative exploration of the readiness of the WHO building blocks, we adapted the framework which was outlined in the context of the SARA tool and the USAID tool ‘Readiness assessment: moving towards a country-led and –financed HIV response for key populations’ [24, 25].

This adapted framework also adapted a semi-structured framework containing several thematic areas to understand the readiness of each building block of the health system in terms of their availability and readiness – including the presence of enablers and challenges at public health system – to ensure providing STI services to the KPs. We considered a component of the building block “ready” if it met all the criteria of the adapted framework, “not ready” if it did not meet any criteria of the framework, and “partially ready” if it met most of the criteria of the framework and required a few areas of improvement. No specific adaptation strategy was adopted, rather, the research team members and co-investigators (both of which include authors of this paper), convened to brainstorm how these two models would be applied for the analysis of the qualitative data. Ideas and feedback were exchanged before the finalization of the adaptation strategy. Then the framework was shared with peer colleagues working with health systems studies under the Health Systems and Population Studies Division of icddr,b. With this consultation, we reached to this dynamic framework entailed looking at the relationship between the actions and actors in the following manner:

**Table:** 

**Building block**	**Key thematic areas**^a^** for exploring the readiness**
Service delivery	Availability of health infrastructure, availability of STI services for KPs in public health facilities, the ways the inputs and services related to STI are organised and managed to ensure availability and accessibility of KPs into public health facilities, maintaining the quality, safety and continuity of care across health conditions and different layers (i.e., primary, secondary and tertiary) of public health facilities, availability of trained and sensitised health workforce, STI service uptake by KPs^b^
Health workforce	Availability, distribution, and performance of health workforce at different layers of the public health system, enablers and barriers of STI service delivery from service provider perspectives
Health information system	Availability of health information structure^c^ at the public health system, option for data capturing and record-keeping for KPs, tracking performance, the process of timely and quality reporting, strengths and improvement areas in the health information system
Medical products/technologies	Availability of STI drugs; the status of equitable access to those drugs by KPs at the public health system; the procurement, supply, storage and distribution systems of health and medical products (i.e., medicine for STI for KPs)
Health financing system	Available financial resources, fund allocation process for a sustainable approach to provide STI services to KPs, scopes for utilising resources, and accountability for supporting STI services to KPs from the public health system
Leadership/ governance	The technical action accompanied by the policy guidance; the process of overseeing and guiding of public health system related to STI services; the functions carried out by the government and leaders of public health facilities (e.g., Hospital Directors, Civil Surgeons) to ensure equitable service access and KPs' rights as patients; the roles and responsibilities of the public, private and voluntary sectors – including civil society – and their relationships with each other in pursuit of ensuring STI services to KPs from the public health system in terms of ensuring a sustainable system for health

^a^In other words, indicators for understanding and exploring the readiness of the public health system.

^b^While SARA focuses on key three indicators (i.e., health infrastructure, health workforce, service uptake), we have incorporated a few key areas alongside these indicators from USAID ‘Readiness assessment: moving towards a country-led and –financed HIV response for key populations’ tool [22]. The indicators had been outlined as ‘key thematic areas for qualitative understanding.’

^c^Health information structure refers to the platform on which health information is stored, and accessed by authorised people [23].

## Methods

### Research team composition

The research team consists of a multidisciplinary assortment of disciplines of expertise, such as anthropology, public health, sociology and clinical medicine. Specifically, the first author (also the Principal Investigator of this study) is an anthropologist with long-term experience of working with KPs. All authors possess considerable experience and expertise in the discipline of HIV and AIDS, in addition to substantial qualitative experience. The non-author researchers were recruited based on their previous experience with qualitative research and underwent rigorous two week-long training about the dynamics of KP communities, health systems and qualitative data collection.

### Study sites

We chose three districts for conducting the study: Dhaka, ChapaiNawabgonj, and Munshiganj. Dhaka was selected because it comprises the three layers of healthcare facilities (i.e., primary, secondary, and tertiary). The reason for selecting Dhaka is that this metropolitan city has all layers of public healthcare facilities (i.e., primary, secondary, and tertiary levels of healthcare), this district has the highest concentration of KPs, and STI/HIV prevention interventions are available for all the KPs in Dkaka district.

Unlike Dhaka, in Munshigonj, there are only primary and secondary levels of public healthcare facilities, which include a district hospital and several primary public healthcare facilities such as upazilla health complex and community clinics. In addition, despite the presence of pockets of KPs, during the study period, there was no STI/HIV prevention intervention for KPs in this district due to the low concentration of KPs in Munshigonj.

We included Chapai Nawabganj district as a study site, which has primary and secondary layers of healthcare facilities. Contrasted to Munshugonj district, there were STI/HIV prevention interventions for all KPs in Chapai Nawabganj district, and services are delivered through three DICs.

### Data collection

Several data collection methods were used in this research to understand the readiness of the public health system in terms of building blocks framework. This dynamic framework allowed us to explore the actors and actions as the building blocks interacting with one another. We conducted 29 key informant interviews (KIIs) using purposive and maximum intensity sampling approaches. At the policy level, the key informants included the Director of Hospitals and Clinics and the Line Director of Hospital Services Management, the Deputy Director of Hospital Services Management, the Director of Primary Health Care Services, and the Director of Finance from the Director General of Health Services Office. In addition, the Director of Logistics and Supply from the Director General of the Family Planning Office, and Director of the Institute of Public Health (IPH), and the Deputy Director (and former Programme Manager, of the AIDS STD Programme) of the Institute of Public Health from the Institute of Public Health (IPH) were interviewed as key informants. From the Tertiary level of the public healthcare facilities, the key informants included the Deputy Director, Head of the Department of Skin and Venereal Diseases (Dermatology), and Associate Professor of Skin and Venereal Diseases (Dermatology) at Dhaka Medical College Hospital, Sir Salimullah Medical College Hospital, and Shaheed Suhrawardi Medical College Hospital. From the Secondary level of the public healthcare facilities, we interviewed the Resident Medical Officers at the Chapai Nawabgonj and Munshigonj District Hospitals. From Primary Healthcare Facilities, we interviewed Resident Medical Officer and Emergency Medical Officer from Upazila Health Complex of Shibgonj Upazila of Chapai Nawabgonj district. From the International NGOs and the UN, we interviewed representatives from UNAIDS, UNICEF, and Save the Children. In addition, five key-informant interviews were conducted with the leaders of Community-Based Organisations (CBO), as well as experienced programme managers and researchers working with a diverse group of KPs.

In addition, we conducted 11 focus group discussions (FGDs) with service providers at DICs not only with the KPs (i.e., MSM, hijra, FSW and PWID) but based on emerging understanding, we conducted a few FGDs with some selected information-rich research participants to understand the experience of KPs about receiving STI services from the public healthcare facilities. In addition, we conducted FGDs with service providers at DICs. In each FGD, 6–8 participants attended from homogenous populations (e.g., MSM, FSW, service providers of DIC, etc.). The participants were approached for interview via phone or email. It is worth noting that there were no pre-existing relationships with the research participants. Moreover, the participants did not know anything about the researchers besides for the reasons for conducting the research. The interviews were conducted at the DICs and the public healthcare facility premises, mainly in the offices of the healthcare providers.

The KIIs lasted approximately 60–90 min whereas the FGDs lasted 90–120 min. To preserve participant confidentiality, no one was present in the interviewing room besides the participant and the researcher(s). The data collection techniques, participant categories and issues explored in each data collection approach is summarized in Table 1.

**Table 1:** Summary of data collection approaches adopted in this study

SL	Data collection approach	Sampling approach	Participant category	Issues explored
1	Focus group discussion (FGD)	Maximum intensity of information	Service providers (including DIC managers, Medical Assistants, Outreach supervisors), and physicians and support staff of public healthcare facilities	Barriers to seeking healthcare at public healthcare facilities, societal perspectives towards public healthcare services, possible ways forward for improving healthcare for these population groups
2	Key informant interview (KII)	Maximum intensity of information	Director/Deputy Director of tertiary hospitals, Civil Surgeons, Director General of Health Services (DGHS) representatives, representatives from UN bodies, participants from Upazila Health Complex, Community-Based Organisations (CBO) leaders, well as experienced programme managers and researchers working with a diverse group of KPs	Existing challenges of the public health system in terms of their preparedness for serving KPs, and recommendations for improving healthcare services for these groups
3	In-depth interview (IDI) (findings published in a separate paper)	Maximum variation sampling	MSM (including MSW), *hijra,* FSW and PWID	Barriers to seeking healthcare, reasons for not visiting the healthcare facilities, recommendations for improving healthcare services
4	Direct observations	-	Public healthcare facilities	The existing public healthcare setup, whether confidentiality is maintained, the existing infrastructure, existing limitations in human resources, etc

Separate sets of semi-structured guidelines were used for the different groups of participants (attached in Additional File 1). The guidelines were developed for the purpose of this study and have not been published elsewhere. These face-to-face interviews were conducted by the first author and a few other co-authors. It is worth noting that no repeat interviews were conducted. The interview guidelines were field tested by the researchers. All of the interviews were recorded upon receiving the informed and understood consents of the participants. On the same day of data collection, the field researchers listened to the audio-recorded interviews, and transcribed the interviews verbatim in a computer. The transcripts were stored in a password-protected computer that could only be accessed by authorized personnel.

Since the qualitative data collection and analysis processes are ongoing and reflexive, various data collection and analysis tools and methods were integrated, thus fulfilling the conventions of methodological triangulation. Moreover, this process allowed the research team to pinpoint areas of saturation. In addition to conducting interviews, the research team (which included physicians, anthropologists and sociologists) also conducted repeated direct observations at public health facilities. The observations were conducted concurrently alongside the interviews. The issues explored in the direct observations are delineated in Table 1. The team documented important observations, researchers’ subjective interpretative findings and any other emerging issues based on guidelines specified by many qualitative researchers. Upon receiving the consent of the participants, the interviews were recorded using a tape recorder. The researchers also documented written field notes immediately after leaving the field. During the FGDs, on the other hand, one researcher was responsible for writing notes on non-verbal communications and other important issues.

### Data analysis

After data collection, the research team rigorously transcribed the interview recordings, while also identifying and analyzing emerging themes and sub-themes that do not fall within the analytical frameworks. The data analysis team consisted mainly of the author researchers, including the first author and the senior author. Rather than using software, they manually analysed the data via line-by-line thematic analysis strategies, without resorting to any data analysis software. The themes that were developed were also based the SARA tool and WHO building blocks. For example, if there was a quotation citing the challenges of the health workforce, this was categorized under the “health workforce” building block. As opposed to assigning themes to the data independently, a group of researchers (including the authors) performed the thematic analysis procedure together. Specifically, before assigning a theme to a particular quotation, the researchers discussed the theme and arrived at a consensus. If there were any disagreements, those were also resolved by the senior members of the research team. Atypical or unique findings were not ignored during the process but rather explored and analysed further.

Moreover, we modified our data analysis scheme where appropriate, after verifying our findings and analysis with research colleagues and participants. To ascertain scientific rigor in qualitative research, and validity and reliability in the broader scientific spectrum, a wide variety of strategies were used throughout the research process, particularly triangulation of two or more data sources, methods, investigators, and approaches to analysis of qualitative data. For example, in terms of data source triangulation, data were collected not only from the healthcare providers but also from members of the KP communities, CBO/NGO representatives, hospital directors, and researchers. Moreover, methodological triangulation has been achieved through adopting a variety of data collection techniques such as IDIs, FGDs, KIIs, and direct observations. Moreover, the data collection has been conducted by a diverse pool of researchers emerging from different backgrounds and levels of seniority. Analytical triangulation has been applied in terms of using both the SARA tool and WHO building blocks for thematically analyzing the data. Peer debriefing was also regularly conducted to facilitate the exchange of findings and interpretations. The insights from the peer debriefing sessions also facilitated the modification of the interview guidelines where appropriate. Furthermore, there was minimal bias as the researchers had long-term experience in conducting qualitative research and were therefore aware of the potential biases.

The study was approved by the Research Review and Ethical Review Committees of the International Centre for Diarrhoeal Diseases Research, Bangladesh (icddr,b) under the ethical approval number of PR-16069. Although participants were entitled to the right to decline participation in the study or withdraw at any time during data collection, none of the participants refused to participate.

## Results

The findings of this qualitative research were thematically analysed using the WHO building blocks framework. Each component of the building blocks showed varying degrees of readiness, which are delineated below. Moreover, other emerging issues were also explored.

### Service delivery readiness

#### Challenges of maintaining privacy in public health system

Physicians of the public health facilities mentioned that it was difficult to maintain privacy for STI patients, including KPs. Our repeated observations also revealed that space constraints in the public health facilities hindered physicians’ abilities to maintain the privacy of the patients who sought treatment for STIs. While senior physicians had their own room, the medical officers typically had to share a room with two or three other colleagues, to maximise the utilisation of limited space. In many cases, these physicians had to see patients concurrently, therefore, more often than not; the patients were in close proximity with fellow patients.

The physicians acknowledged that the space limitations in the public health facilities hindered STI patients’ abilities to non-hesitantly disclose personal information with the physician. They also acknowledged that as STI patients from general population struggle to freely communicate their concerns due to the crowded environment, the struggle might be even more pronounced for KPs because their practices are generally not accepted by society. KP anecdotes have confirmed this struggle, while FSW, for example, noting that they felt humiliated while trying expressing STI related symptoms, since other patients stood next to them.

In addition, observations indicated that the patient queues frequently overflow into the physicians’ rooms. Therefore, patients were also within hearing range of the conversation between the physician and the patient who was currently being seen. The setting as a whole was not favourable for conducting physical examinations of STI patients because patient privacy was being compromised. Despite physicians’ efforts to regulate the queues, patients persisted to step into the room or peak through the door while the patient is being examined. Some physicians attribute this problem to the paucity of support staff, such as Members of Lower Support Service (MLSS) to ensure that queues were maintained in an orderly manner.

However, some physicians felt so constrained for time and space that they could not even allot a bed and timeframe for conducting a physical examination despite the presence of a bed and curtains. In turn, this impedes privacy and confidentiality. In the interest of saving time, the physician sometimes instructs their patients to expose themselves in front of their desk itself, thus inadvertently making them visible to the other patients. As the physicians from a tertiary setting explained:

Sometimes we have to treat so many patients that taking each patient behind the curtain for examination seems impossible and appear to be a waste of time! In these situations, if there is no other patient in the room other than the doctors, we tell them to take off their clothes just there. What else can we do? (Medical Officer from Skin and VD department of tertiary healthcare facility, KII)

It is challenging for us to conduct physical examinations for patients requiring physical examinations to be conducted in the lying position because there is no bed [in that specific room]. For the female patients, we need to use a focused light but unfortunately; we do not have any fixed light source. This is a matter of maintaining the privacy. Since we cannot conduct the examinations here, we take the patients to the operating room, which has a bed and fixed spotlight. (Consultant of from Skin and VD department of tertiary healthcare facility, KII)

#### Physicians’ limited awareness about the hijra and MSM

According to the findings, the facilities were not deemed to be ready for providing services for the MSM and *hijra* because they were not aware of the complexities that could be affecting these populations, thus affecting their ability to render gender-responsive services for them. Despite the recognition of *hijra* as a separate gender category, many physicians are grossly uninformed about the biological and psychosexual characteristics of *hijra*. Many of them assume that *hijra* suffer from a “genetic disorder”, thus giving rise to their androgynous appearance. Some physicians even recommended that the government conduct a screening test to determine their “actual gender identity.” A few *hijra* visiting public health systems also recounted the following mortifying experience:

We were in the waiting room and one of my *hijra* friends was in the ultrasound room. There were many other people along with a few *hijra*. Suddenly, a junior doctor came to this room and said that the patient is not an “original *hijra*” and he has a male organ. He also said to us, “why did you not reveal earlier that he has penis and testes?” This was showing disrespect and humiliation towards us. We felt ashamed about this statement in front of others. Most people do not know that being a *hijra* is not only biological but also psychiatric. (*Hijra*, KII, Dhaka)

Most physicians also mentioned feeling anticipatory fear around *hijra*. A female physician, for example, expressed that, “they [*hijra*] carry both male and female organs, and I was anxious about how to deal with her and how she might act around my chamber.” Other instances had illustrated the physicians’ limited understanding about the sexual behaviour of *hijra* and MSM had culminated in them developing misconceptions about their patients. For example, some physicians perceive male-to-male sex as a psychiatric “problem” that warrants psychotherapeutic measures; hence they referred the MSM to the psychiatry department of the same facility to “correct” the behaviour.

To ensure the KPs’ accessibility to STI services, healthcare providers need to interact with KPs in a respectful, considerate and culturally sensitive manner. However, across the districts and all the layers of public healthcare facilities, they described the behavior of KPs as *onoitik achoron* (meaning “immoral behaviour”) and *ossavabik* (meaning “abnormal”). These sentiments manifested themselves in the way they treated the KPs. Referring to MSM, some physicians mentioned “homosexuality is a psychological problem which needs to be cured” while, a few physicians indicated that same-sex relations are criminalized within the legal framework of Bangladesh thus MSM and *hijra* need to be punished, rather than getting treatment. Hence, they felt intimidated and wanted to minimize contact with them. For instance, one of the physicians of Chapai Nawabganj district hospital explained:

Once, I treated a *hijra* patient. I have seen that they are usually clapping in their typical style and asking for money. This experience made me scared and uncomfortable in their presence. Therefore, whenever I see a *hijra* patient, I try to show the least interest in her … to get rid of her as quickly as possible. (Medical Officer, secondary healthcare facility, KII)

#### Lack of history-taking due to physicians’ understanding of KPs’ behaviour and time constraints

An increased understanding of KPs could eliminate any history-taking-related barriers, which were challenging for physicians dealing with STIs. Ideally, the physician would have ample time to learn the underlying reasons behind their patients’ problems through history-taking. However, we found that this was not possible due to heavy patient inflow during the study period. Besides, limited understanding about the KPs also made it difficult for the physicians to take the history of KPs seeking treatment for STIs. A public health expert and physician explained that not taking the complete patient history could lead to misdiagnosis, incorrect conclusions, or a missed diagnosis if the physicians overlook important information due to lack of time and knowledge limitations about health issues which KPs could be vulnerable to. He explained that:

Taking complete histories is essential for patients with STIs, particularly KPs. If we do not take complete histories, it could lead to missed diagnosis in terms of STIs, like the misdiagnosis of gonorrhea, for example. Another example can be asymptomatic STIs which the doctor could easily miss. Also, misdiagnoses could occur in the sense that the doctors mistake gonorrhea for UTI if the patient does not give the exposure history. (Physician and public health expert, KII)

#### Counseling: the missing component

Counseling is an essential tool that supports people to make their own informed decisions about safer sex, partner management, STI risk reduction, and assist KPs to improve their self-esteem. Therefore, counseling is a key facet in the holistic healthcare model of healthcare, in which addressing psychological issues are crucial for patient management. Most of the physicians opined that counseling should be incorporated as a preventive measure because it involves behaviour change communication, where counselors could empower patients to make informed decisions about their own health and well-being. Physicians also acknowledged the importance of counseling but noted that “due to the huge patient flow in government hospitals, we can only prescribe medicines and give brief advice, which we cannot even elaborate on” (Medical Officer, Skin and VD department, DMCH, KII). There was no post for Counselor at any of the public health facilities during the study period, even though several key-informants opined that there should be at least one counselor deployed at each primary healthcare facility.

#### Attitudes and behaviours of health care providers (HCP) towards KPs

To ensure health service accessibility, HCP and other Human Resource for Health (HRH, such as Physician/Doctor, Nurse, Technologist, Storekeeper, Pharmacist, Ward boy, Guard, Cleaner, MLSS) would need to interact with KPs in a respectful, considerate and culturally sensitive manner. However, they described KPs’ behaviours as *onoitikachoron* (meaning “immoral behaviour”) and *ossavabik* (meaning “abnormal”). These sentiments manifested themselves in the way they treated the KPs. They also mentioned feeling apprehensive about KPs, fearing that they would be a “nuisance” in public health facilities. Hence, they feel intimidated and want to minimise contact with them. For instance, one of the physicians of Chapai Nawabgonj district hospital explained:

I once got a *hijra* patient in my private chamber. I have seen that they are usually clapping in their typical style and asking for money. … And this experience made me feel scared and uncomfortable around them. Therefore, when I saw the *hijra* patient, I tried to seem least interested in her … and tried to get rid of her as quickly as possible. (Medical Officer, secondary healthcare facility, KII)

Similarly, some HCP developed negative impressions and felt uneasy about PWID because of their “tendencies to steal others’ valuables and cause a commotion.” One physician even mentioned that they “stop them from [loitering in the hallways] [and] ask them to leave the hospital when their treatment is over” (RMO, DMCH, KII). Another physician explained that nurses and ward boys often do not want to treat the PWID patients “due to their unhygienic condition and malodor” therefore they “try to stay away from the PWID as much as possible” (Assistant Professor, DMCH, KII). KPs also noted the unwillingness of staff to touch them; therefore they have to manage their own problems. He explained that:

I visited the government hospital. Everyone was trying to avoid me. The [nurse/ward boy] did not want to come nearby me due to my abscess’s bad odor, despite persistent requests. No one came forward to dress my abscess. I was asked to manage it myself on the veranda (balcony). (PWID, 25 years old, FGD, Dhaka)

#### Unavailability of STI testing in primary layer public health system

To ensure STI health services for KP, health facilities need to be equipped with the appropriate diagnostic tools for the etiological management of STIs. Findings have revealed that there are provisions for STI testing, such as Venereal Disease Research Laboratory (VDRL) and Treponema Palladium Hemagglutination Assay (TPHA) tests in all the public health facilities studied, but not at the primary layer of the public health system.

### Health workforce readiness

The findings of this study indicated that while all the positions allocated for the public health system were posted, the existing human resource for health (HRH) was insufficient in proportion to the ever-increasing patient flow in the studied facilities. Although the medical officers are primarily responsible for seeing the initial cases, senior physicians also have to share this responsibility due to the substantial patient influx. A medical officer explained that:

It looks like this hospital is running by the mercy of Almighty Allah. A few months ago, 8-9 doctors were appointed here and I was relieved because I thought we had a lot of doctors only to find out that they soon transferred out. And then we fall into the same crisis yet again. (Resident Medical Officer, secondary healthcare facility, KII)

Physicians who are well-acquainted and sensitised with the lifestyles of KPs were rare. However, we noticed a few of these physicians being transferred during the study period, Hence, if this type of physician were to transfer from their area; the KPs would have to undergo the hassle of developing the same rapport with another physician.

Physicians at Chapai Nawabgonj and Munshigonj mentioned their preference of moving to Dhaka or practicing at the divisional cities instead. In addition, MLSS and nurses prefer practicing in their hometowns, thus leading to staff shortages in divisional city facilities. MLSS are particularly helpful because they could help regulate patient queues and prevent patient overflow into the physicians’ room. The physicians noted that there is no recruitment procedure or circular for support staff. A director member of a tertiary medical college hospital reported that, “after 2010, no new MLSS were appointed and whatever MLSS was here before is no longer here because some of them retired or transferred to other locations.”

The primary and secondary health facilities do not have Skin and VD departments; therefore, the medical officers are delegated the responsibility of seeing the STI patients. However, female STI patients who came to the outdoor facilities are referred to the Gynecology & Obstetrics department or the patients go there themselves. Nevertheless, the existing medical officers still struggle to cope with the increasing inflow of patients. Discussions with physicians revealed that there is a shortage of specialised physicians at the peripheries because the physicians prefer practicing at divisional cities instead. Hence, deploying Skin and VD consultants at peripheral cities is challenging. As a result, it is difficult for KPs, particularly those who live in remote areas, to attain the specialised healthcare that they need. It is highly unlikely that the medical officers or other specialists would be sensitised and informed about KP lifestyles and practices in ways that can address KPs’ needs.

### Readiness of medical products/technologies

#### Availability of STI drugs to treat KPs

The studied hospitals in Dhaka have all the STI medicines, barring two (for syphilis and herpes). The hospitals in the other districts are missing two additional medicines although there are alternative medicines, albeit for general populations. For example, the second-line drug Benzyl Benzoate is kept for treating scabies, instead of first-line drug Permithrine Cream. Since STI patient inflow is low, hospital authorities decided not to keep Permithrine Cream in the medicine stock. While hospitals expressed interest, pharmaceutical companies did not want to participate in the tender “if the requested number is low, because it will more likely lead to a financial loss for them” (Senior in-charge, SSMCH, KII). Findings indicate that many drugs (first-line or second-line) were available at public health facilities to treat STIs for KPs. However, some drugs were needed for STIs, especially antibiotics, therefore healthcare facilities must ensure all of these drugs for STI treatment.

#### Availability of medical technologies to treat KPs

Medical technologies such as examination equipment and instruments are essential diagnostic tools. Vaginal speculum and proctoscope are crucial for vaginal and anal examinations, respectively, yet they are available at the Skin and VD department. One of the physicians explained this scenario as such:

Instruments for examination are not enough. Mainly a few of the instruments required for gynecological examination are not available, such as vaginal speculum. We have to refer these patients to the department of Gynecology & Obstetrics for examination purposes. Then these patients get treatment from that department. (Medical Officer, Dept. of Skin and VD of a tertiary healthcare facility, KII)

Nevertheless, hospital directors reassured that if the inflow of patients, particularly KPs, suffering from STIs increases in the Skin and VD department, these instruments can be arranged with approval from the higher authority. They stated that these approvals may take time, but advocacy from the bilateral organisations at the Ministry and Directorate General office may help expedite the approval of these resources.

### Readiness of health information systems

Across the three layers of the public health system, patients’ health information is recorded, stored, and presented via a web-based health information systems (HIS) software named District Health Information System, version 2 (DHIS2). This data allows for data entry in addition to the instant generation of summary tables, charts and GIS maps. Our key informants indicated that this health information management scheme is internationally acclaimed as the “best practice” in LMIC by several international donors and experts such as WHO, GIZ and PATH.

However, the current HIS strategy has some areas for improvement to capture detailed data on the KPs. Our key informants noted that recording patient histories is difficult in the existing setup. These challenges might be difficult to overcome quickly, as mentioned by the physicians and experts working at DGHS. At the outdoor department, only three types of information are documented on a daily basis: the total number of patients on that day, their sex and age group (using a pre-determined age range). At the secondary level, this procedure is maintained at the ticket counter. At the end of the day, they circulate this information to the statistics department. On the other hand, at the indoor department, the information that is inscribed at the outdoor facilities is also recorded, in addition to the monthly-updated disease profile data set. It is important to note that there is no provision to indicate a third gender in the database or the manual ticket counters, despite *hijra* legally being recognised as a third gender.

Higher-level directors noted that recording detailed histories could be difficult in the existing setup, primarily because separate, specialised software is required. While the interface of the software was not an issue, it would be challenging to maintain the software through human resources. For instance, separate health workforce is needed to log the patient details. It is not possible for the physician to shoulder this responsibility because it is already difficult to manage time to see a huge number of patients, let alone set aside time for data entry. Hence, it would be more pragmatic to allocate separate staff members for data entry. However, the directors noted that they already struggle with a staff shortage, so allocating separate staff for data entry would not be feasible at this moment.

Moreover, a workable Internet connection is required for storing data on the server. In addition, a hospital-wide connection will facilitate the interconnectedness of various departments within the facility. For instance, if the laboratory had internet access, the relevant staff members could automatically upload the laboratory investigation results onto the database. According to the Head of Pathology of DMCH, it would be difficult for them to implement this system due to the paucity of trained health workforce and technological support.

### Readiness of health financing system

While directors claimed that funds can be arranged for providing treatment and ensuring the privacy of KPs, one of the directors posited that a substantial amount of financial investment is necessary for procuring and maintaining the hardware for information preservation for maintaining the health information system. To run the software, a viable device is needed in the form of a desktop, laptop, tablet or Smartphone. Yet, it is difficult to procure these devices within the current budget.

However, these gaps can be overcome, if the government assumes the responsibility of treating the KPs, which would entail allocating additional funds to the Operational budget. The hospital directors were amenable and ready to use funds to improve STI treatment for patients suffering from STIs, including the KPs. However, sufficient funds have to be dedicated to this sector and utilised in a timely manner.

### Leadership and governance

The functionality of each component of the healthcare facilities is governed by the leaders in the public health system. In order for government health facilities to be conducive to treatments for STI patients and KPs, health service providers need to be adequately trained and sensitised. Several directors and senior physicians pointed out that HCPs’ limited awareness about KPs could potentially hamper treatment, which indicates their awareness of existing challenges and openness to overcome those. As one of the Directors, for example, explained:

Most people generally do not have a positive view of *hijra*. We [physicians] are no exception and we are part of this society. The physicians usually see them how the remainder of the general population sees them. In a lot of cases, general people try to stay away from *hijra*. That also means physicians could have the same feeling. Therefore, it is important to increase the awareness about *hijra*, explaining to the doctors that *hijra* are human beings too and we can’t marginalise them. It’s also important to facilitate separate training for the physicians who would be dealing with them. (A hospital management senior official, KII)

However, before ensuring that HCPs are sensitised and informed about the cultural and sexual practices of KPs, it is important to ensure that there are a sufficient number of physicians who can comfortably manage STI cases. According to most of the directors, it would be preferable to at least deploy a Skin and VD consultant at all layers of the public health system, if not arrange for a separate Skin and VD department.

One of the directors proposed a mechanism to incentivise physicians to serve at facilities at the peripheries. He said that the physicians serving at the peripheral duty stations could be offered higher salaries proportionate to their distance from the capital. However, this is a policy decision, which can be considered by the government. Moreover, he mentioned that the government is planning to recruit more than 10,000 physicians at the different levels of healthcare, which could alleviate the staff shortage.

Overall, the leadership of different layers of the public health system was open to improving different areas of the public health system to ensure treatment of STIs in a KP-friendly manner.

## Discussion

This study revealed that in general, the public health system was not ready to serve the needs of the KPs in terms of providing quality STI services. The ‘service delivery’ which is one of the most important building blocks of the public health system was unready to provide STI services to KPs in Bangladesh. However, a few components such as health workforce and health, information systems, etc. were partially ready although these components need to be systematically addressed to increase its readiness.

A major part of understanding the service delivery readiness is exploring the readiness of physicians and other healthcare providers to administer health services to KPs. Despite admitting feeling uncomfortable in the KPs’ presence, they are nonetheless amenable to the idea of treating KPs because they feel like it is their inherent duty as a physician. This principle was implemented in only a few countries, such as north-east India where they integrated women who inject drugs into the public health system [26]. However, the majority of the literature in developed and developing countries, still indicated that the healthcare providers were unwilling to provide basic STI services to these population groups [27–29].

Nevertheless, many physicians from this study mentioned that PWID can sometimes cause commotion in the healthcare setting and steal others’ valuables, therefore they try to minimise contact with them as much as possible. Similarly, Neale et al.’s studies reflected similar sentiments among the PWID patients [30]. Moreover, our PWID interviews not only revealed that healthcare providers tried to refrain from contact with them but also exhibited antagonising behaviours. On the other hand, while PWID are easy to identify, it is even easier to distinguish a *hijra* due to their distinct appearance. Some physicians have encountered *hijra* outside the healthcare setting, and generally feel apprehensive toward them. In addition, the healthcare providers from this study seemed to possess limited awareness about the anatomical and psychosocial characteristics of *hijra*, thus placing the *hijra* patients in unpleasant situations. Findings from a qualitative study on the *hijra* in Bangladesh echoed similar findings that the physicians seemed to possess little to no understanding of *hijra* culture and it seemed to affect the way they treated the *hijra* patients [31]. Studies in several other countries showed similar barriers [32–34]. Moreover, The perceptions of male-to-male sex being a psychological problem were corroborated by various studies worldwide. For instance, a study based in Bosnia revealed one particularly striking quote, explaining that the physicians were suggesting a “cure” for the MSM patients, such as seeing a religious preacher or trying to cure “homosexuality” [35]. On the other hand, however, studies based in Europe showed high disclosure rates of MSM while availing STI services. An online multi-country sexual health survey revealed that disclosure rates of sexual practices ranged to as high as 94% in London and had a median of 73% [36]. Thus, this signified the readiness of these healthcare facilities to render STI screening and treatment.

Another essential component of effective service delivery is by maintaining privacy and confidentiality. However, the findings revealed that maintaining confidentiality and privacy in the healthcare setting is extremely challenging because of space constraints and overflowing patient queues. While there is evidence from other LMIC-based studies depicting the paucity of privacy and confidentiality, there is no previous study that has delineated the underlying reasons for the government health facilities’ struggle to retain privacy, more so the impact of these barriers on KPs’ uptake of STI services public health system. However, a cross-sectional study in the same region depicted that privacy was being upheld for patients, which is a stark contrast to our study findings. In particular, the survey demonstrated that 81.4% of the patients in the public health facilities were accorded a certain degree of privacy. On the other hand, however, complete informational confidentiality was maintained only in 10.8% of the public health facilities [37].

In addition to maintaining privacy and confidentiality, history-taking is also challenging because, due to the limited awareness of the physicians, there could be a missed diagnosis or misdiagnosis. There is substantial evidence corroborating the findings from this study, portraying the effect of limited understanding on their history-taking abilities. Though Joarder et al.’s study is focused on the general populations, the findings have revealed the challenges that physicians face while taking patient histories [38]. The study mentioned that physicians seemed to listen attentively to the patients but at the expense of compromising time spent on physical examinations. Moreover, the physicians are constrained for time which could be spent giving detailed advice to patients, which also correlates to the argument in this study noting that physicians do not have enough time to counsel their patients therefore they merely offer brief advice. However, in this study, it seemed as though the physicians neither had time for listening to the patient nor conducting private physical examinations. Both the studies demonstrated that there is limited scope for history taking in the public health system, which might produce considerable challenges for KPs. According to an analysis by McKay, disclosure of sexual history can be a paradoxical “catch-22” situation because the inability to disclose sexual orientation or identity could undermine healthcare whereas disclosing these details could exacerbate these populations’ healthcare access barriers [39]. These findings are also applicable to the KPs in this study because interview findings revealed that physicians regarded them differently as soon as they revealed sexual identities. This statement is particularly true if the physicians are not particularly informed about KP lifestyles and practices, because they would not be able to use contextual cues to infer their patients’ health issues.

The study noted some challenges in the HRH situation in the facilities that were studied. Because of the low physician-to-patient ratio, physicians struggle with the ever-increasing patient flow, despite investing considerable efforts. The patient flow also hinders the physicians’ ability to dedicate adequate time for giving advice to the patients, let alone counseling. A recent study shows that the average consultation length for patients in Bangladesh is 48 s, and short consultation length is likely to “adversely affect patient care and the workload and stress of the consulting physician” [40].

Studies including this one highlighted the importance of highly qualified human and sensitised HCP and HRH to provide STI services to the KPs, and that Bangladesh has a chronic shortage of health workforce, particularly in the peripheries [41]. This study also illustrated that it seems as there are occupied positions for Skin and VD consultants, but the turnover rates lack promise. Moreover, there is a severe shortage of MLSS which was more prominent in the major cities, which is also reflected by national health data. The underlying contexts of turnover are yet to be reflected in other local research, despite the concerning implications of this situation. Therefore, the findings of this study were able to bridge this pressing knowledge gap.

Many of the drugs for treating STIs are available at public health facilities. Despite the unavailability of first-line drugs to treat syphilis and herpes, second-line drugs were available for treating KPs with STIs. Findings indicated that drug availability is solely demand-based, therefore hospitals do not typically stock on low-demand medicines. In contrast, a qualitative study based in Kenya revealed that STI drug stock-outs were common, thus hindering the quality of STI services for PLHIV [42]. Most importantly in the context when many antibiotics for treating STIs are becoming resistant and there is no surveillance on antibiotic sensitivity, availability of proper antibiotics is necessary. While some medical technologies are available for STI treatment, some physical examination instruments are in short supply, because the technologies are not available in their own departments. This complexity is yet to be explored in other research. Likewise, Islam and Biswas’s study noted that while there are problems with the effective supply chain management, scarcity of funding or inability to release timely funds could engender serious implications [3].

In terms of health information systems, at the indoor and outdoor facilities alike, it was reported that keeping the record and store detailed history of KPs can be challenging given the existing setup, due to the limitations in trained health human resources, logistics and technological resources. However, this can be manageable if sufficient funds and HRH are allocated. On the other hand, a literature review conducted in 43 countries revealed that the most pressing barrier in maintaining electronic health information systems is the resistance to change among healthcare providers [43]. Several studies noted that capacity-building initiatives would be beneficial for bridging these knowledge limitations. However, there remains a lack of research that analyses the limitations of health information systems using a health assessment framework.

This study revealed that there were budgetary allocations for various components of the public health system such as equipment (e.g., curtains, beddings, lighting) and capacity-building training for physicians about KPs. However, key-informants almost unanimously informed that ensuring government allocation and procurement is always challenging and higher officials need to be involved. However, the equipment can be procured if an additional allocation can be managed through the existing procurement management system. Nonetheless, this study participants stated that the major challenge of healthcare financing is the “bureaucratic delays” which delay in fund disbursement and utilisation. Therefore, the top structural level of public health systems needs to be involved to address this problem, to ensure sustainable service delivery for KPs. These structural-level complexities were yet to be explored in other research. Rather, the literature emphasized healthcare avoidance among these population groups due to personal financial constraints and lack of health insurance coverage [44–46].

One of the core constituents of health systems strengthening is ensuring a robust leadership and governance strategy. The findings of this study revealed that higher authorities of the public health system are open to supporting this initiative, but structural advocacy initiatives are needed to address existing resource limitations in several areas of the health system. However, on the other hand, some of the studies showed that not all the health management and governance structures at public health systems did not exhibit a similar tone of support, thus compromising the acceptability of healthcare for LGBT populations [47, 48]. Thus, prioritisation is critical which may come to fruition through continued advocacy. Most importantly, the non-judgmental attitudes of the high-level management need to be ensured for improving the health system so that it is more conducive not only for STI services but also overall services targeted for KPs. Incentive opportunities could be introduced to alleviate the physician shortage at the peripheries, by offering higher compensation. Moreover, to relieve the overall physician deficit on the national level, the government is planning to recruit more than 10,000 physicians at the various layers of healthcare. All the Hospital Directors and Deputy Directors emphasised that it would be helpful to facilitate capacity building and sensitising initiatives to train doctors, nurses and other healthcare providers to enter relevant data and render KP-friendly services.

### Study limitations

Qualitative research is not designed to generalize the findings. Thus, readers’ expectations of generalization would not be fulfilled by this study. Rather this study has provided an in-depth understanding of the context. Since this study was conducted in three districts, thus, the scenario of other districts of Bangladesh cannot be generalized. However, because of the trustworthiness of qualitative data, there will be similarities in the context of other districts. Moreover, as the public healthcare facility structure differs across these three districts, we were able to elicit a diversified picture of the healthcare-seeking scenario of Bangladesh.

### Implications of the findings and future directions

This study indicated that the various layers of the public health system in Bangladesh showed varying degrees of readiness to administer STI services to KPs. There are scopes for improving service delivery, health human resources, and health information system. A few components such as medical products and technologies, the healthcare financing system, and the public health system’s leadership and governance can be ready with the help of systematic advocacy for resource allocation can be taken. While the government can initiate the improvement of the different building blocks of health systems to make them reasonably ready to provide STI services to the KPs, preventive services (such as community-based outreach services) would be difficult to be implemented by the Government.

In this context, the Public Private Partnership (PPP) model or a framework needs to be applied by involving NGOs and CBOs. We also recommend these study findings be used to pilot synergistic interventions to facilitate integrative preventive and curative services for STIs. If communities are not encouraged and engaged, they are unlikely to visit even a ready health facility. This is not possible to exclude the NGOs/CBOs overnight from the preventive services. Moreover, it is essential to include KP communities in the health systems strengthening process. By gaining emic insights from their perspective about the ways in which health systems can be improved according to their needs, it would be easier to tailor health services to respond to their complexities. Such approaches have demonstrated promising outcomes in other countries. For example, a review article synthesized over 70 qualitative studies which explored the struggles of black MSM throughout the US, some of which were transposed into culturally sensitive structural interventions to respond to their healthcare needs on the national health facility level [49]. Similarly, community mobilization efforts for KPs instituted by a renowned intervention called Avahan have refined the healthcare response for these population groups by sensitizing healthcare professionals, educating participants about the importance of their health, etc. [50]. Therefore, community engagement is an essential ingredient for informing a sustainable healthcare system.

While the NGOs, CBOs, and KP communities are involved in community mobilisation/community engagement, and encourage the KPs to attend the public health facilities, the government needs to arrange skilled and culture-sensitive human resources, appropriate medicines, equipment, and logistics for running these services, thus creating a complementary effort through PPP framework. Most importantly, this framework must be tested with rigorous and appropriate research methodologies to generate evidence-based potential models which can be implemented effectively. Before such a model is tested and proven with efficacy/efficiency with cost-effectiveness, rapid transitioning to government health systems may run the risk of jeopardising the success of HIV interventions which has been achieved for over 20 years in Bangladesh.

## Conclusion

The AIDS/STD Control Programme of the Government of Bangladesh is planning that STI services for the KPs to be successfully rendered by the government health systems. In order to have a successful transition, public health systems need to be fully ready. However, as described above public health facilities are yet to be fully ready to render STI services to KPs, especially in terms of service delivery and human and health resources. Therefore, it is not only integral to mobilize communities towards the uptake of public health services, but at the same time, public health systems need to be prepared to cater to their needs. To promote a sustainable transition from NGOs to public health facilities, some service delivery components need to be improved such as ensuring the availability of a culturally sensitised health workforce, making counseling services available and addressing STI service limitations at primary layers of healthcare facilities.

### Supplementary Information

**Additional file 1.** English interview guidelines- consists of various semi-structured questionnaires for different groups of informants such as health service providers of DICs and public healthcare facilities, government officials, and CBO leaders. The guidelines also contain a guideline for consultation workshops and an observation checklist for the public healthcare facilities.

**Additional file 2.** Consolidated criteria for reporting qualitative research (COREQ) – consists of the characteristics of the qualitative methods section for this manuscript.

## Data Availability

It is not possible to publicly share the data set generated and/or analysed during the current study at this moment. Sharing the data may position the study participants at risk due to existing punitive laws against MSM and PWID in Bangladesh right at this point of time. For instance, according to the revised Narcotic Control Act of 2018 in Bangladesh, certain clauses prohibit the carrying, use, and possession of drugs and drug paraphernalia therefore disclosing information about the PWID participants who engage in these activities could potentially incur legal repercussions. Likewise, since male-to-male sex is a punishable offense as per Bangladesh Penal Code 377, disclosing their sex practices could lead to similar legal implications. For these reasons, the authors are not willing to publicly share their data. However, upon reasonable request, the corresponding author might share data after a certain period as per data policy of icddr,b.

## Abbreviations

ASP: AIDS/STD Control Programme
CBO: Community-based organisation
DHIS2: District Health Information System, version 2
FSW: Female sex worker
FGD: Focus group discussion
HCP: Healthcare providers
HIS: Health information systems
HIV: Human immunodeficiency virus
HRH: Human resources for health
IDI: In-depth interview
KII: Key-informant interview
KP: Key population at risk of HIV
LMIC: Low and middle income countries
MSM: Men having sex with males
MSW: Male sex worker
NGO: Non-governmental organisation
PPP: Public private partnership
PWID: People who inject drugs
SARA: Service availability and readiness assessment
STI: Sexually transmitted infections
