# Correction to: Are we ready for a sustainable approach? A qualitative study of the readiness of the public health system to provide STI services to the key populations at risk of HIV in Bangladesh

**DOI:** 10.1186/s12913-023-10093-7

**Published:** 2023-10-09

**Authors:** Gorkey Gourab, Golam Sarwar, Mohammad Niaz Morshed Khan, A M Rumayan Hasan, Samira Dishti Irfan, Tarit Kumar Saha, Lima Rahman, A. K. M. Masud Rana, Sharful Islam Khan

**Affiliations:** 1 Programme for HIV and AIDS, Infectious Diseases Division, International Centre for Diarrhoeal Diseases Research, 68 Shaheed Tajuddin Ahmed Sarani, Mohakhali, Dhaka, 1212 Bangladesh; 2 Graduate School of Biomedical Sciences, Hiroshima University, Hiroshima, Japan; 3 Universal Health Coverage, Health System and Population Studies Division, International Centre for Diarrhoeal Diseases Research, 68 Shaheed Tajuddin Ahmed Sarani, Mohakhali, Dhaka, Bangladesh; 4 Institute of Public Health (IPH), Dhaka, Bangladesh; 5 Ministry of Health and Family Welfare, 68 Shaheed Tajuddin Ahmed Sarani, Mohakhali, Dhaka, Bangladesh; 6 HIV/AIDS Programme, Health, Nutrition and HIV/AIDS Sector, Save the Children, House 35, Road 43, Gulshan-2, Dhaka, Bangladesh

**Correction to:** ***BMC Health Services Research*** **(2023) 23:979**

10.1186/s12913-023-09996-2

Following publication of the original article [1], the authors identified an error caused by a typesetting mistake in the author name of the fourth author.

The incorrect author name is: AMRumayan Hasan

The correct author name is: A M Rumayan Hasan

The author group has been updated above and the original article [1] has been corrected.
