# Supplementary material for: Are we ready for a sustainable approach? A qualitative study of the readiness of the public health system to provide STI services to the key populations at risk of HIV in Bangladesh
Source: BMC Health Serv Res. 2023 Sep 11;23:979. doi: 10.1186/s12913-023-09996-2 (PMC10496154; doi:10.1186/s12913-023-09996-2)
Supplement: Supplementary file 1 — Additional file 1. English interview guidelines- consists of various semi-structured questionnaires for different groups of informants such as health service providers of DICs and public healthcare facilities, government officials, and CBO leaders. The guidelines also contain a guideline for consultation workshops and an observation checklist for the public healthcare facilities. [file 12913_2023_9996_MOESM1_ESM.docx]

## English guideline for observations at public healthcare facilities

## Presence of the KPs at the public health facilities

## Behaviour of the hospital staff towards KPs

## Interactions of KPs with people involved in service delivery

## Reaction of the general patients in presence of the KPs

## Dress-up of the hijra and other KPs at public healthcare facilities

## Identifying the gatekeepers at public healthcare facilities

## English guideline for focus group discussions for service providers at the Drop-in Centres (DICs)

## Do you believe the public/government health system is ready to provide STI related services to KPs? Please explain your reasoning.

- What are the barriers and enablers for KPs to receive STI-related services from these facilities? Please explain in terms of the following healthcare components.
  - Availability of appropriate services for addressing STI concerns
  - Privacy and confidentiality of services
  - Respectful interactions with the healthcare service providers
  - Knowledge among healthcare providers about community dynamics of KPs and their healthcare concerns.
  - Availability of medicines and equipment for STI related concerns

## How ready is the community to receive STI-related services from public health facilities? What are their experiences from the public healthcare facilities?

- We have heard that the KPs prefer seeking care from the DICs as opposed to the public healthcare facilities? Based on your experience, why they do prefer DICs?
- What services from the DICs do you think would be beneficial for them if incorporated into the public healthcare setting?
- What can be the possible ways to overcome the challenges and how their positive experiences can be utilized to receive STI-related services from public health facilities?

## What can be the possible ways for a functional integration between government and non-government sectors in relation of KP-related services?

- What services can be provided from the public healthcare facilities for the KPs? Which services should be continued with the current modality?
- Who are the key stakeholders who should be involved in this process?

##

## English guideline for key informant interviews with service providers at public/government healthcare facilities

**KII Guideline for Service Providers**

| **ID** | **Type of KI** | **Age** | **Education** | **Sex** | **Occupation** | **Area** | **Remarks of interviewer** |
| --- | --- | --- | --- | --- | --- | --- | --- |
|  |  |  |  |  |  |  |  |

**Selection criteria of the key informant:**

| **Specific Objectives** | **Guiding questions** |
| --- | --- |
| 1. To understand the **readiness of leadership and governance** at public/government health systems to provide STI related services (i.e., prevention and treatment) to the key populations (KP) at risk of STI and HIV transmission | What do you think about the KPs who are at risk of STI transmission?  The functions carried out by the government hospitals and other public healthcare facilities for STI-related services?  How you are planning to improve population health while ensuring equity in access to services?  How the quality of services related to STI is ensured?  What is your standpoint of patients' rights?  What is your standpoint to provide STI-related services to KPs who are at risk of STI and HIV transmission?  In your view, what are the roles and responsibilities of the public sector in pursuit of national health goals (STI-related services to KPs as a component)? |
| 1. To understand the **readiness of health financing system** at public/government health systems to provide STI and HIV related services (i.e., prevention and treatment) to the key populations (KP) at risk of STI and HIV transmission | - Do you think that the public/government health system is ready with adequate funds to support STI services for KPs? - Do you think additional funding will be required if you want to provide STI-related services to the KPs? If “yes”, why do you think so? How this funding can be managed? |
| 1. To understand the **readiness of the public health facilities in terms of medical products/technologies** at public/government health systems to provide STI related services (i.e., prevention and treatment) to the key populations (KP) at risk of STI and HIV transmission | - Is the STI-related medicines are available at the public/government health facility? - Is the public/government health facility ready with medical products/technologies required for the management of STIs if KPs come to receive these services from these facilities? - How the stock is maintained? - Was there any stock out situation of STI medicines in recent years? - What might be the challenges in ensuring the equitable access to medical products/technologies required for the management of STIs of KPs? What are the enablers? |
| 1. To understand the **service delivery readiness** at public/government health systems to provide STI related services (i.e., prevention and treatment) to the key populations (KP) at risk of STI and HIV transmission | - What services, related to STI, are available for KPs? - Are those services easily accessibility by KPs? - What are the potential barriers and facilitating factors influencing access to STI-related services by KPs? |
| 1. To understand the **health workforce readiness** at public/government health systems to provide STI related services (i.e., prevention and treatment) to the key populations (KP) at risk of STI and HIV transmission | - Are there sufficient and trained human resources available to provide STI-related services to KPs? - Are they sensitized to deliver STI-related services to KPs in a non-judgmental manner? - What are the service providers' experiences and perceptions about various KPs? What do they think about these populations? - To you, what are the barriers to deliver STI-related services to KPs? What are the enablers? - What are the issues related to health workforce those may appear as barriers (e.g. staff turnover)? |
| 1. To understand the **health information system readiness** at public/government health systems to provide STI related services (i.e., prevention and treatment) to the key populations (KP) at risk of STI and HIV transmission | What is the current data recording and reporting system?  How the data about the KPs receiving STI-related services will be recorded and reported?  What are the strengths and areas for improvement in current health information system? |
| *Not all questions are appropriate for all groups (e.g. Director and deputy director at government hospitals, Director and deputy director of public health facilities, Directorate General of Drug Administration (DGDA), Directorate General of Health Services). However, it is expected that the interviewers will select the right questions for right people during interviews.* | |

**English guideline for key-informant interviews of government officials**

1. To understand the **readiness of leadership and governance** at

public/government health systems to provide STI and HIV related services (i.e., prevention and treatment) to the key populations (KP) at risk of STI and HIV transmission

- What do you think about the KPs who are at risk of STI and HIV transmission?
- What are the functions carried out by the government hospitals and other public healthcare facilities for STI-related services?
- How you are planning to improve population health while ensuring equity in access to services?
- How the quality of services related to STI is ensured in public health facilities?
- What is your standpoint about patients' rights?
- What is your standpoint to provide STI-related services to KPs (who are at risk of STI and HIV transmission) from public health facilities?
- In your view, what are the roles and responsibilities of the public sector to execute national health goals (STI-related services to KPs as a component)?
- Do you think your existing management is capable to provide STI-related services to KPs?

2. To understand the **readiness of health financing system** at public/government health systems to provide STI and HIV related services (i.e., prevention and treatment) to the key populations (KP) at risk of STI and HIV transmission

- Do you think that the public/government health system is ready with adequate funds to support STI services for KPs?
- Do you think additional funding will be required if you want to provide STI-related services to the KPs? If “yes”, why do you think so? How this funding can be managed? From when it can be possible to start?

3. To understand the **readiness of the public health facilities in terms of medical products/technologies** at public/government health systems to provide STI-related services (i.e., prevention and treatment) to the key populations (KP) at risk of STI and

HIV transmission

- Is the required STI-related medicines are available at the public/government health facility?
- Is the public/government health facility ready with medical products/technologies required for the management of STIs if KPs come to receive these services from these facilities?
- How you ensure supply chain management of medicine and other medical products
- How the stock is maintained?
- Was there any stock out situation of STI medicines in recent years?
- What might be the challenges in ensuring the equitable access to medical products/technologies required for the management of STIs of KPs? What are the enablers?

4. To understand the **service delivery readiness** at public/government health systems to provide STI and HIV related services (i.e., prevention and treatment) to the key populations (KP) at risk of STI and HIV transmission

- What services, related to STI, are available for KPs?
- Are those services easily accessible by KPs?
- What are the potential barriers and facilitating factors influencing access to STI-related services by KPs?

5. To understand the **health workforce readiness** at public/government health systems to provide STI services (i.e., prevention and treatment) to the key populations (KP) at risk of STI and HIV transmission

- Are there sufficient and trained human resources available at different tier of government health facilities to provide STI-related services to KPs?
- In which areas do the existing government human resources need capacity building?
- Are they sensitized to deliver STI related services to KPs in a non-judgmental manner?
- What are the service providers' experiences and perceptions about various KPs? What do they think about these populations?
- To you, what are the barriers to deliver STI-related services to KPs? What are the enablers/Strengths?
- What are the issues related to health workforce those may appear as barriers (e.g. staff turnover)?
- How they would overcome the challenges maintaining required confidentialities?

6. To understand the **health information system readiness** at public/government health systems to provide STI-related services (i.e., prevention and treatment) to the key populations (KP) at risk of STI and HIV transmission

- What are the current data recording and reporting system at different layer of govt. health facilities?
- How the data about the KPs receiving STI-related services will be recorded and reported?
- What are the strengths and areas for improvement in current health information system?

## English guideline for key informant interviews with leaders of community-based organizations for KPs

- Tell me about your long-term experiences working with these populations
- For these populations, we are hoping to transition them from the current HIV programs to the existing public and private healthcare facilities. What are your opinions about this issue? What types of challenges do you think they would face? How can we overcome these challenges? Please explain based on your experience
- If we could incorporate any services from the HIV prevention intervention model into the healthcare setting, which ones would be introduce and why?
  - Would it be possible to introduce testing and treatment for STIs?
  - What about the distribution of condoms and lubricants?
  - What about abscess management?
  - What about counselling?
- We have heard about some negative experiences from these populations about their experiences at the public healthcare facilities. We are aware that there are community mobilization initiatives within the HIV intervention program to motivate them to visit these facilities but why are they still suffering from these negative experiences?
  - How are these negative experiences preventing them from getting the healthcare that they need?
  - What is a possible way out for this situation? What are your possible recommendations?
  - A lot of people mentioned that doctors and support staff are not sensitized about these populations, so who can we involve in sensitization initiatives for these healthcare providers?
  - On the other hand, a few participants have reported positive experiences about the healthcare facilities. How can we utilize these good experiences to motivate your community members to uptake services from these facilities?
- Let’s say we were to design a public private partnership model for integrating healthcare services for these populations. Please describe what the model could look like?
