# Supplementary material for: Are we ready for a sustainable approach? A qualitative study of the readiness of the public health system to provide STI services to the key populations at risk of HIV in Bangladesh
Source: BMC Health Serv Res. 2023 Sep 11;23:979. doi: 10.1186/s12913-023-09996-2 (PMC10496154; doi:10.1186/s12913-023-09996-2)
Supplement: Supplementary file 2 — Additional file 2. Consolidated criteria for reporting qualitative research (COREQ) – consists of the characteristics of the qualitative methods section for this manuscript. [file 12913_2023_9996_MOESM2_ESM.docx]

**S1 Table. COREQ checklist (1)**

For:

**Are we ready for a sustainable approach? A qualitative study of** **the readiness of the public health system to provide STI services to the Key Populations at risk of HIV in Bangladesh**

| **Topic** | **Item No.** | **Guide Questions/Description** | **Details/ reported on page no.** |
| --- | --- | --- | --- |
| **Domain 1: Research team and reﬂexivity** | | | |
| *Personal characteristics* | | | |
| Interviewer/facilitator | 1 | Which author/s conducted the interview or focus group? | Gorkey Gourab  Mohammad Niaz Morshed Khan  AM Rumayan Hasan  Golam Sarwar  Page no. 6 (Methods, Paragraph 4) |
| Credentials | 2 | What were the researcher’s credentials? E.g. PhD, MD | Gorkey Gourab (GG), MSS, MPH  Mohammad Niaz Morshed Khan (MNMK), MSS, MPhil, MPH, PGD  AM Rumayan Hasan (AMRH), MSS, MPH  Golam Sarwar (GS), MBBS, MPH, PGD  Samira Dishti Irfan (SDI), MPH  Tarit Kumar Saha (TKS), MBBS  Lima Rahman (LR), Master in Population and Reproductive Health Research, MBBS  AKM Masud Rana (AKMMR), PhD  Sharful Islam Khan (SIK), MBBS, MHSS, PhD  Non-author data collectors held Master’s degree and above.  Page no. 5 (Methods, Paragraph 1) |
| Occupation | 3 | What was their occupation at the time of the study? | GG, MNMK, AMRH, GS, SDI, AKMMR and SIK were full time researchers with many years of experience in the field of HIV and AIDS. GS and SIK are licensed physicians. The rest of these authors are anthropologists, social scientists and public health experts. All of them worked at International Centre for Diarrhoeal Disease Research, Bangladesh (icddr,b) and were also involved in implementing intervention on HIV for the key populations (KP) at risk of HIV.  TKS worked for the National AIDS/STD Programme for the Government of Bangladesh. LR worked on HIV/AIDS Program at Save the Children, USA. TKS guided the National response to HIV and AIDS in Bangladesh, and LR worked on implementing intervention on HIV for KP. Both of them had many years of research and program implementation experience in the field of HIV and AIDS.  Non-author data collectors were full-time researchers who also had experience with HIV and AIDS related issues and key populations.  Page no. 5 (Methods, Paragraph 1) |
| Gender | 4 | Was the researcher male or female? | Gorkey Gourab (GG), male  Mohammad Niaz Morshed Khan (MNMK), male  AM Rumayan Hasan (AMRH), male  Golam Sarwar (GS), male  Samira Dishti Irfan (SDI), female  Md. Masud Reza (MMR), male  Tarit Kumar Saha (TKS), male  Lima Rahman (LR), female  AKM Masud Rana (AKMMR), male  Sharful Islam Khan (SIK), male  Among non-author data collectors, two were female and two were male.  Page no. 5 (Methods, Paragraph 1) |
| Experience and training | 5 | What experience or training did the researcher have? | GG, MNMK, AMRH, GS, SDI, SIK: Extensive qualitative research experience on HIV and AIDS among key populations (KPs).  AKMMR, TKS, LR: Experience on quantitative and mixed method research, key populations, HIV and AIDS.  Non-author data collectors were recruited based on their prior experience on qualitative research, and received ten-day long training on qualitative data collection and analysis, community and health system readiness, HIV and AIDS.  Page no. 5 (Methods, Paragraph 1) |
| *Relationship with participants* | | | |
| Relationship established | 6 | Was a relationship established prior to study commencement? | No, no relationship has been established between researchers and research participants.  Page no. 6 (Methods, Paragraph 3) |
|  | 7 | What did the participants know about the researcher? e.g. personal goals, reasons for doing the research | They did not know anything except for reasons for conducting the research, which was described to the research participants to receive written or verbal consent.  Page no. 6 (Methods, Paragraph 3) |
| Interviewer characteristics | 8 | What characteristics were reported about the inter viewer/facilitator? e.g. Bias, assumptions, reasons and interests in the research topic | We do not believe that there were any biases significant to the report, since the researchers were fully aware about the potential bias and had long experience of conducting qualitative research. All the researchers had ethics certification on conducting research on human subjects; and GG, MNMK, AMRH, GS, AKMMR, SDI and SIK were certified on Results-based management (RBM). Besides, the research team followed several mechanisms to minimize bias of qualitative data collection and analysis such as triangulation, peer-debriefing, etc.  Page no. 5-6 (Methods, Paragraphs 1 and 6) |
| **Domain 2: Study design** | | | |
| *Theoretical framework* | | | |
| Methodological orientation and Theory | 9 | What methodological orientation was stated to underpin the study? e.g. grounded theory, discourse analysis, ethnography, phenomenology,  content analysis | In our study, we aimed to explore and understand the readiness of the public healthcare facilities to render STI services to key populations at risk of HIV. For analyzing qualitative data, we followed thematic and contextual analysis techniques. We also applied the theoretical proposition where we integrated a qualitative adapted version of the Service Availability and Readiness Assessment (SARA) tool and the healthcare building blocks by WHO.  Page no. 6 (Methods, Paragraph 5) |
| *Participant selection* | | | |
| Sampling | 10 | How were participants selected? e.g. purposive, convenience,  consecutive, snowball | The participants were purposively selected following the maximum intensity sampling procedures to elicit in-depth information from information-rich participants.  Page no. 6 (Methods, Paragraph 3) |
| Method of approach | 11 | How were participants approached? e.g. face-to-face, telephone, mail,  Email | Focus group participants were selected though service centers, known as Drop-In Centers (DIC), which the research team has been implementing for over a decade. Key-informants were contacted for interview via telephone or email. Face-to-face interviews were conducted.  Page no. 6 (Methods, Paragraph 4) |
| Sample size | 12 | How many participants were in the study? | We conducted 29 Key-Informant Interviews (KII) with Director/Deputy Director of tertiary hospitals, Civil Surgeons, Director General of Health Services (DGHS) representatives, representatives from UN bodies, participants from Upazila Health Complex, as well as experienced program managers and researchers working with diverse groups of KPs. Besides, we conducted 11 focus group discussions (FGDs) with service providers at DICs.  Page no. 6 (Methods, Paragraph 3) |
| Non-participation | 13 | How many people refused to participate or dropped out? Reasons? | No participant refused to participate.  Page no. 7 (Methods, Paragraph 7) |
| *Setting* | | | |
| Setting of data collection | 14 | Where was the data collected? e.g. home, clinic, workplace | Data was collected from different layers of public healthcare facilities (i.e., primary, secondary and tertiary). Data was also collected at DICs from the service providers.  Page no. 6 (Methods, Paragraph 4) |
| Presence of non-  participants | 15 | Was anyone else present besides the participants and researchers? | No, no non-participant was present besides the participants and researchers.  Page no. 6 (Methods, Paragraph 3) |
| Description of sample | 16 | What are the important characteristics of the sample? e.g. demographic  data, date | Participants were selected through maximum intensity sampling approach.  Page no. 6 (Methods, Paragraph 3) |
| *Data collection* | | | |
| Interview guide | 17 | Were questions, prompts, guides provided by the authors? Was it pilot tested? | Yes, semi structured interview and FGD guidelines were field tested by the researchers. In addition, considering the emerging nature of qualitative data, the research team interpreted and exchanged findings through peer-debriefing meetings, which helped us modify data collection guidelines as needed.  Page no. 6 (Methods, Paragraph 6) |
| Repeat interviews | 18 | Were repeat inter views carried out? If yes, how many? | None.  Page no. 6 (Methods, Paragraph 4) |
| Audio/visual recording | 19 | Did the research use audio or visual recording to collect the data? | Interviews and FGDs were recorded using tape recorders where the participants gave verbal/written consent to record the interviews. Recordings of interviews and FGDs were transcribed line-by-line on the same day of data collection.  Page no. 6 (Methods, Paragraph 5) |
| Field notes | 20 | Were ﬁeld notes made during and/or after the interview or focus group? | The researchers wrote field notes immediately after the key-informant interviews. During FGDs, while one researcher facilitated the FGD, one researcher was resent to take notes on non-verbal communications and other important field notes.  Page no. 6 (Methods, Paragraph 5) |
| Duration | 21 | What was the duration of the inter views or focus group? | Each interview took one hour to one and a half hours. The duration of FGDs varied from one and a half hours to two hours.  Page no. 6 (Methods, Paragraph 3) |
| Data saturation | 22 | Was data saturation discussed? | Yes, data saturation was discussed during the research process. We stopped data collection at the point of data saturation and point data of redundancy.  Page no. 6 (Methods, Paragraph 5) |
| Transcripts returned | 23 | Were transcripts returned to participants for comment and/or correction? | N/A |
| **Domain 3: analysis and ﬁndings** | | | |
| *Data analysis* | | | |
| Number of data coders | 24 | How many data coders coded the data? | GG, MNMK, AHRH, GS, SDI and SIK coded the data for this research project.  Page no. 6 (Methods, Paragraph 6) |
| Description of the coding  tree | 25 | Did authors provide a description of the coding tree? | Structural coding method by developing joint or collaborative coding framework was followed. Coding tree was not discussed, but it is available upon request.  Page no. 6 (Methods, Paragraph 6) |
| Derivation of themes | 26 | Were themes identiﬁed in advance or derived from the data? | Themes have been derived from the data, but the theoretical frameworks that we used (i.e. SARA and WHO health building blocks) guided us in developing the themes.  Page no. 6 (Methods, Paragraph 6) |
| Software | 27 | What software, if applicable, was used to manage the data? | Qualitative data analysis software was not used to manage the data.  Page no. 6 (Methods, Paragraph 6) |
| Participant checking | 28 | Did participants provide feedback on the ﬁndings? | N/A |
| *Reporting* | | | |
| Quotations presented | 29 | Were participant quotations presented to illustrate the themes/ﬁndings?  Was each quotation identiﬁed? e.g. participant number | Yes, the participant quotations were added and identified.  Page no. 7-14 (Results section) |
| Data and ﬁndings consistent | 30 | Was there consistency between the data presented and the ﬁndings? | Yes, there is a consistency between the data and findings.  Page no. 7-14 (Results section) |
| Clarity of major themes | 31 | Were major themes clearly presented in the ﬁndings? | Yes, they were presented in the findings.  Page no. 7-14 (Results section) |
| Clarity of minor themes | 32 | Is there a description of diverse cases or discussion of minor themes? | Yes, there is such description.  Page no. 7-14 (Results section) |

**Developed from:** Tong A, Sainsbury P, Craig J. Consolidated criteria for reporting qualitative research (COREQ): a 32-item checklist for interviews and focus groups. *International Journal for Quality in Health Care*. 2007. Volume 19, Number 6: pp. 349 –357.
